# Supplementary material for: Towards High Surface Area α-Al2O3–Mn-Assisted Low Temperature Transformation
Source: Materials (Basel). 2023 Apr 12;16(8):3047. doi: 10.3390/ma16083047 (PMC10142860; doi:10.3390/ma16083047)
Supplement: Supplementary file 1 [file materials-16-03047-s001.zip › materials-2323824-supplementary.pdf]

Materials

Supporting Information

Towards High Surface Area α-Al_2O_3–Mn-Assisted Low Temperature Transformation

Tim Jähnichen [1,*], Simon Carstens [1], Maximilian Franz [1], Otto Laufer [1], Marianne Wenzel [2], Jörg Matysik [2] and Dirk Enke [1]

[1] Institute of Chemical Technology, Leipzig University, Linnéstr. 3, D-04103 Leipzig, Germany
[2] Institute of Analytical Chemistry, Leipzig University, Linnéstr. 3, D-04103 Leipzig, Germany
* Correspondence: tim.jaehnichen@uni-leipzig.com

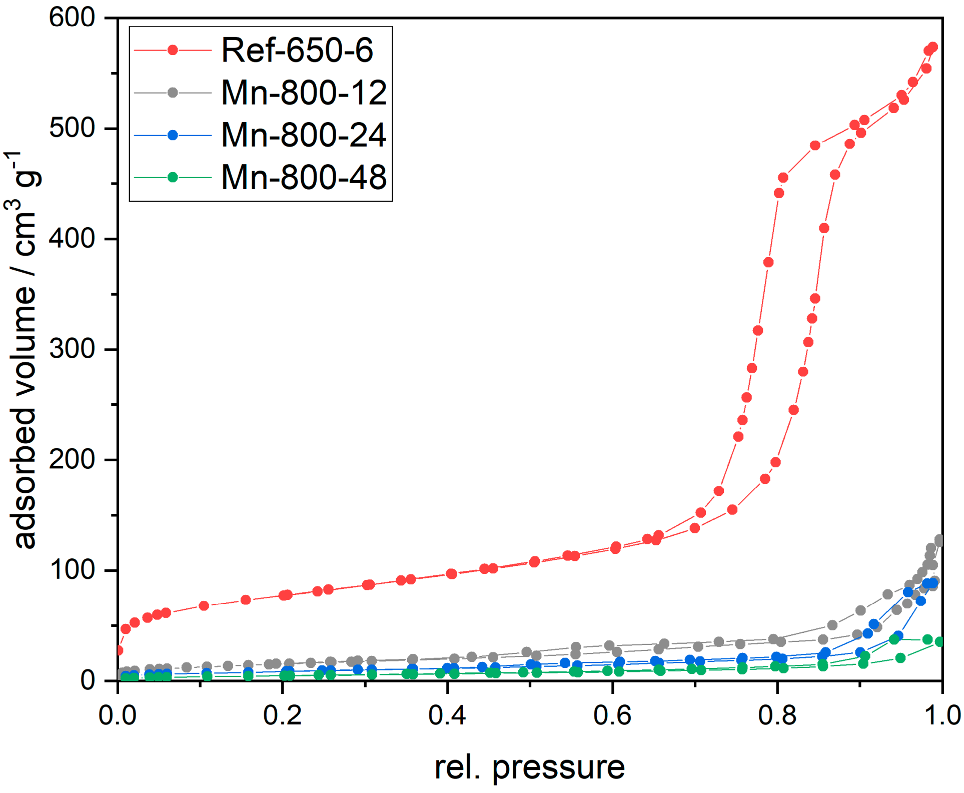

Figure S1. Nitrogen sorption isotherms of the starting material and after calcination at 800 °C for various time periods.

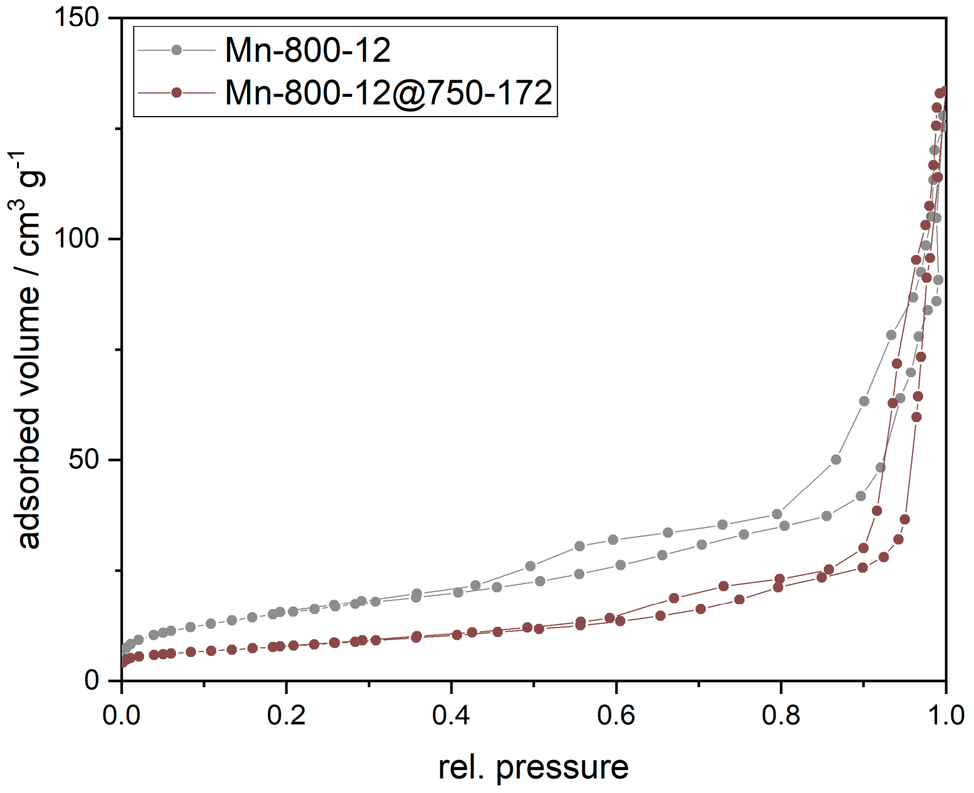

Figure S2. Nitrogen sorption isotherms of Mn-800-12 before and after heat treatment.

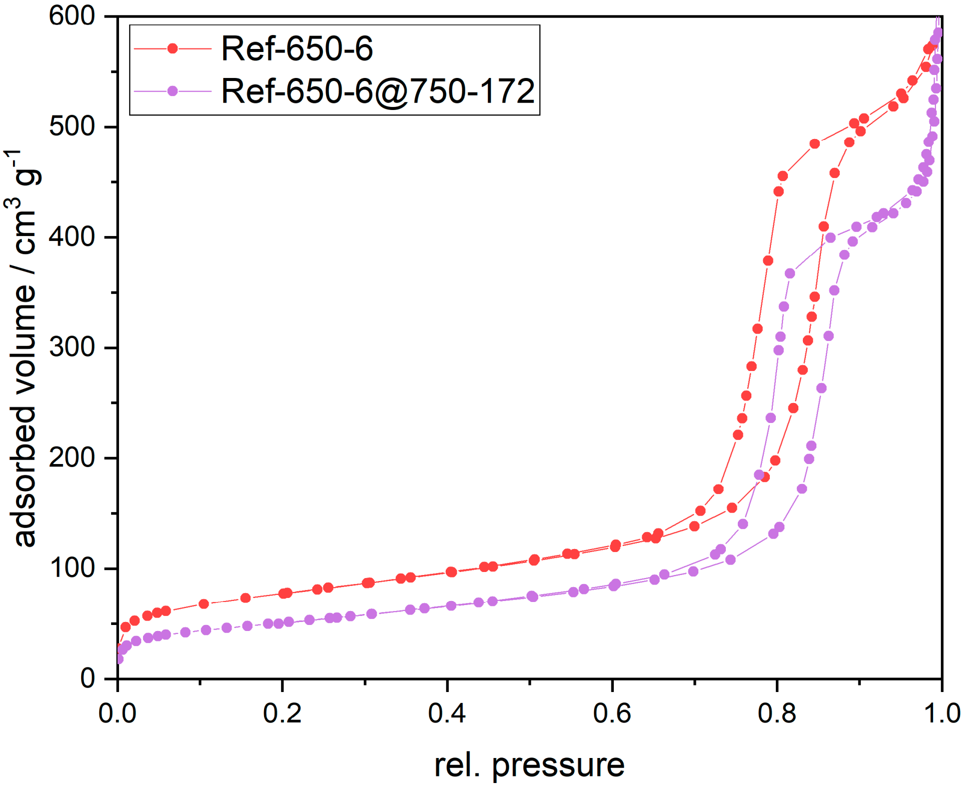

Figure S3. Nitrogen sorption isotherms of Ref-650-6 before and after heat treatment.

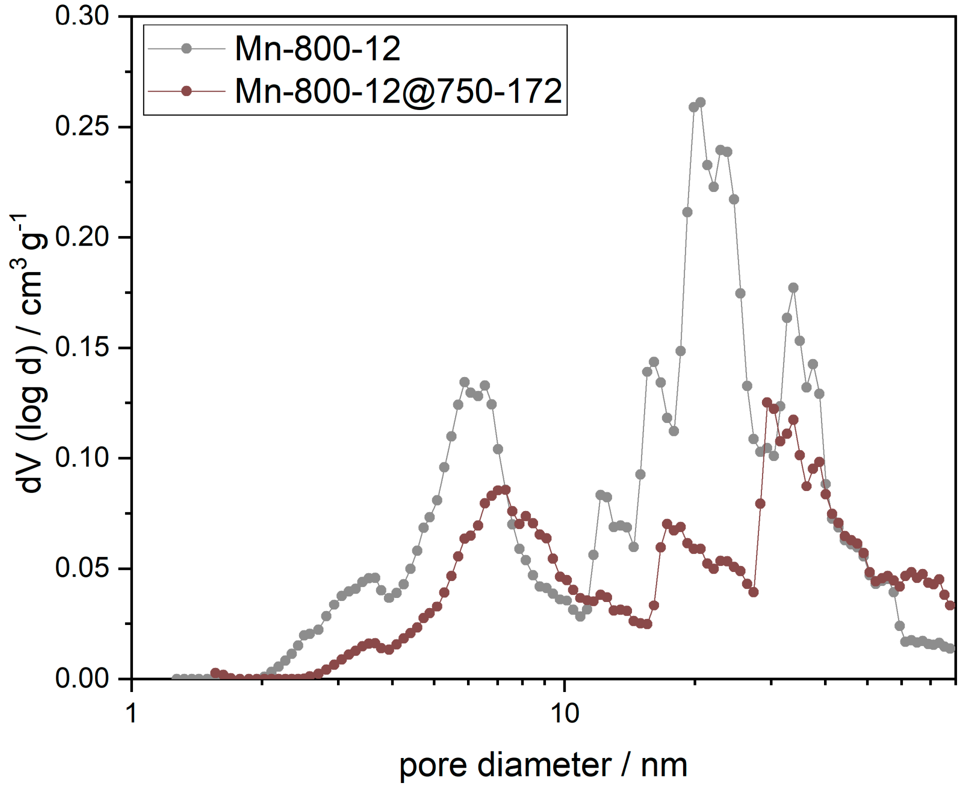

Figure S4. Pore size distribution of Mn-800-12 before and after heat treatment. Calculated from nitrogen sorption.
